# Longitudinal anatomical and visual outcome of macular telangiectasia type 2 in Asian patients

**DOI:** 10.1038/s41598-023-46394-4

**Published:** 2023-11-02

**Authors:** Kiyoto Totsuka, Shuichiro Aoki, Takahiro Arai, Kodai Kitamoto, Keiko Azuma, Ryosuke Fujino, Tatsuya Inoue, Ryo Obata

**Affiliations:** 1 Department of Ophthalmology, The University of Tokyo Graduate School of Medicine, 7-3-1 Hongo, Bunkyo-Ku, Tokyo 113-8655 Japan; 2 Department of Ophthalmology and Micro-Technology, Yokohama City University, 4-57 Urafune, Minami-Ku, Yokohama, Kanagawa 232-0024 Japan

**Keywords:** Eye diseases, Eye manifestations

## Abstract

Limited information regarding the anatomical and visual prognosis of macular telangiectasia (MacTel) type 2 in the Asian population is currently available. Herein, we conducted a retrospective longitudinal analysis of Japanese patients diagnosed with MacTel type 2. Disease progression was evaluated using the Simple MacTel Classification developed by Chew EY et al. in 2023, and its association with visual changes was analyzed. Sixteen eyes of eight Japanese patients were included in the study, with an average follow-up period of 8.2 ± 3.9 years (range, 2.2–14.0). At the initial visit, 7 (44%) and 5 (31%) eyes were classified as Grade 2 (central ellipsoid zone break) and Grade 3 (noncentral pigment), respectively. The proportion of eyes that progressed by 1 or 2-steps in grade after 1, 3, 5, 8, and 12 years was 0%, 14%, 43%, 70%, and 100%, or 0%, 7%, 7%, 30%, and 75%, respectively. The visual acuity significantly deteriorated during the follow-up period, particularly in the two eyes with full-thickness macular holes (FTMH). Three out of 7 patients exhibited low serum serine concentrations, although no apparent correlation with anatomical or visual outcomes was observed. Overall, this cohort demonstrated chronic disease progression, both anatomically and functionally, in eyes with MacTel type 2, with FTMH potentially associated with greater visual loss.

## Introduction

Macular Telangiectasia Type 2 (MacTel) is a rare progressive degenerative disorder that predominantly affects individuals aged 40–60 years and involves the bilateral macular retina^1,2^. The estimated prevalence of MacTel ranges from 0.0045 to 0.1%^3,4^ The typical retinal pathology of MacTel begins in the parafoveal temporal region and extends superiorly and nasally, often presenting with grayish retinal opacities, crystalline-like retinal deposits, and right-angle venules. As the disease progresses, neovascularization, lamellar hole, and full-thickness macular hole (FTMH) can occur^2,5^. Characteristic anatomic findings include dilated parafoveal retinal capillaries with fluorescein leakage on fluorescein angiography, as well as useful imaging tools such as fundus autofluorescence (FAF) and blue light reflectance imaging (BRI) for assessing retinal involvement in MacTel. Optical coherence tomography (OCT) often reveals intraretinal cavities, sub-internal limiting membrane (ILM) spaces, downward displacement of inner retinal layers, and the loss of the inner/outer segment junction^6^.

Recent insights have revealed that degenerative changes in Müller cells underlie the pathology of MacTel^7^. Additionally, studies have implicated the accumulation of neurotoxic metabolite, deoxysphingolipids, in the retina, which is associated with low levels of serine and glycine in the retina^8^.

Previous reports on the long-term course of MacTel have indicated that choroidal neovascularization (CNV), although rare, can lead to significant visual impairment. Even cases without CNV have been associated with milder visual decline^2,9,10^. More recently, pigmentation and ellipsoid zone (EZ) loss have been recognized as important factors in visual impairment and have been incorporated into a simplified classification system^11^.

It is important to note that previous reports have primarily focused on cohorts of Caucasian individuals, with limited information available regarding Asian populations. Only a few studies, such as a cross-sectional study^12^ or a short-term observation within 2 years^13^, have explored MacTel in Asian populations.

In this report, we present a longitudinal analysis of visual acuity and retinal morphology progression in Japanese patients with MacTel, with an average follow-up duration of 8 years. The objectives of this study are to investigate whether MacTel cases in our cohort exhibit progression according to the simplified classification system and to explore potential racial differences in the characteristics of progression.

## Results

The study included sixteen eyes of eight Japanese patients, consisting of three men and five women. Table 1 presents the demographic and systemic characteristics. The mean age at the initial examination was 62.5 ± 7.2 years (range, 53–70). Out of the patients, two (25%) had diabetes, one (13%) had hypertension, and three (38%) had dyslipidemia. The mean follow-up time was 8.2 ± 3.9 years (range, 2.2–14.0). Table 2 summarizes the visual and anatomical findings. Supplementary Figure S1 displays fundus photographs and OCT B-scans with infrared fundus images using Spectralis OCT for both eyes in cases 1–8.

**Table 1:** Demography and laboratory data.

#	Age	Sex	Diabetes	Hypertension	Dyslipidemia	Triglyceride (mg/dL)	Total-cholesterol (mg/dL)	LDL-cholesterol (mg/dL)	Serine (nmol/ml)	Glycine (nmol/ml)	Alanine (nmol/ml)	Beta-alanine (nmol/ml)
1	70	F	+		+	43	162.0	82	108.0	182.8	393.3	2.2
2	70	F				276	253.0	175	123.5	189.9	393.4	4.5
3	61	M		+		134	213	130	118.4	196.2	460.0	1.7
4	65	M				128	220	126	122.2	293.9	495.1	3.0
5	53	F	+		+	199	221	137	**81.9L**	223.3	501.5	2.5
6	56	F			+	**222H**	194	125	**82.2L**	168.8	590.2	1.4
7	53	M				**369H**	209	91	**83.0L**	205.0	511.1	1.7
8	68	F				NA	NA	NA	NA	NA	NA	NA

Significant values are in bold.

**Table 2:** Ophthalmology data.

Eye	SphEq(D)	Follow-up period(yr)	LogMAR			Grade^$^		MNV		FTMH	
			Initial	Final	Change	Initial	Final	Initial	Final	Initial	Final
1R	2.13	14.0	0.10	0.22	0.12	2	3				
1L	2.25	14.0	0.10	0.22	0.12	2	4				
2R	3.50	9.7	− 0.08	− 0.08	0.00	3	4				
2L	3.25	9.7	− 0.08	− 0.08	0.00	3	3				
3R	− 1.25	7.9	− 0.08	− 0.08	0.00	0	0				
3L	0.00	7.9	0.30	0.40	0.10	2	3				+^#^
4R	1.00	5.2	− 0.08	− 0.08	0.00	0	0				
4L	0.00	5.2	0.52	0.70	0.18	2	2				
5R	− 0.50	13.3	0.52	0.52	0.22	2	2	Nonexudative	Nonexudative		
5L	− 0.50	13.3	0.30	0.52	0.22	2	4	Nonexudative	Nonexudative		
6R	0.50	2.2	− 0.08	0.05	0.12	3	3				
6L	0.75	2.2	− 0.08	− 0.08	0.00	0	0				
7R	− 1.88	5.4	0.05	0.22	0.18	0	2				
7L	− 1.50	5.4	− 0.08	0.00	0.08	2	2				
8R	0.25	8.0	0.00	0.15	0.15	3	4				
8L	0.63	8.0	0.30	1.00	0.70	3	3			+	+
Ave		8.2	0.09	0.23	0.14*						

*SphEq* spherical equivalent. *LogMAR* logarithm of minimum angle of resolution. *MNV* macular neovascularization. *FTMH* full-thickness macular hole.

^#^The eye underwent surgery for FTMH.

*p = 0.008 using mixed model.

^$^Grade was determined based on Simple Classification System^11^.

### Disease progression

**Figure 1:**
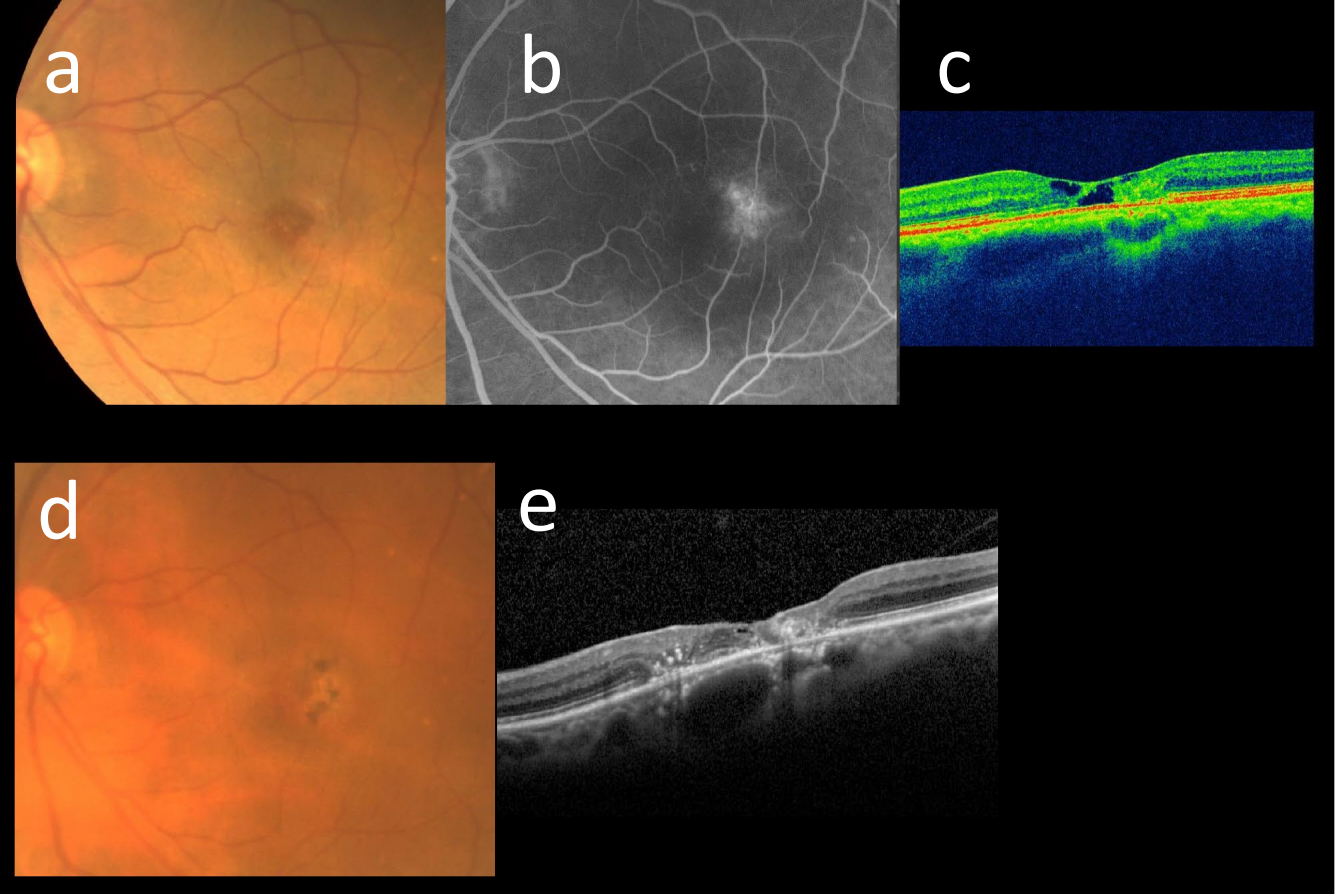
Representative case of Matel type 2 in the current cohort. Seventy-year-old woman (Case 1) presented with visual impairment. Best-corrected decimal visual acuity in the left eye was (0.8). Fundoscopy revealed grayish change in the temporal macula (**a**), showing hyperfluorescence in the fluoresceine angiography (**b**). Ellipsoid zone loss involving the center with inner and outer intraretinal cavity were detected OCT, corresponding to Grade 2 (**c**). After 14 years, noncentral hyperpigmentation was seen (**d**). In OCT, hyperreflectivity at the outer retina was observed (**e**). Decimal visual acuity mildly decreased to (0.6).

**Figure 2:**
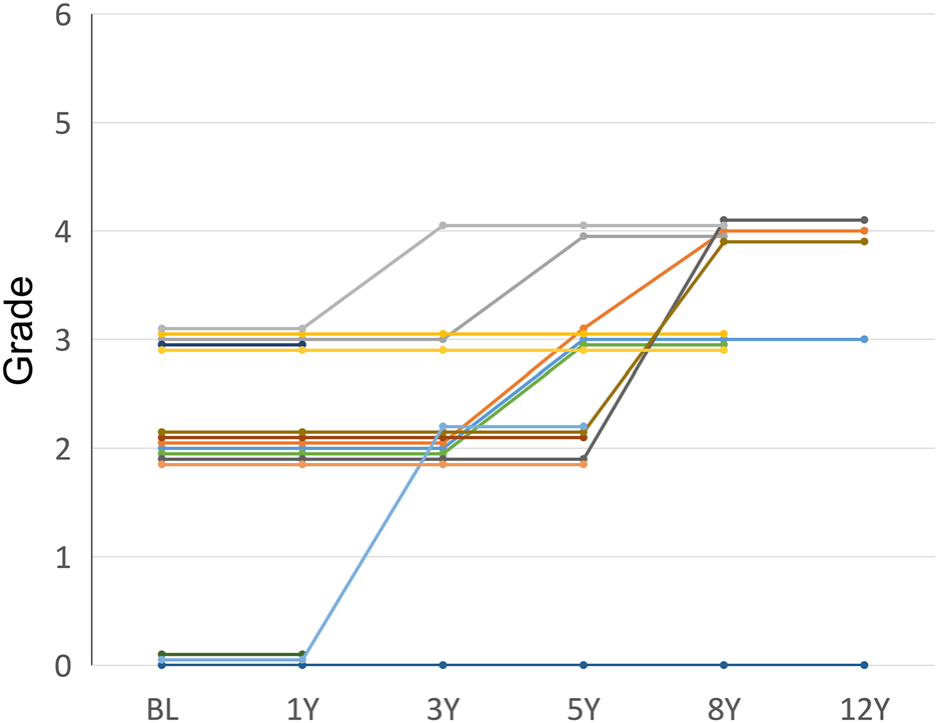
The grade in Simple Classification System^11^ of each eye (n = 16) at the baseline, 1, 3, 5, 8, or 12 years.

**Figure 3:**
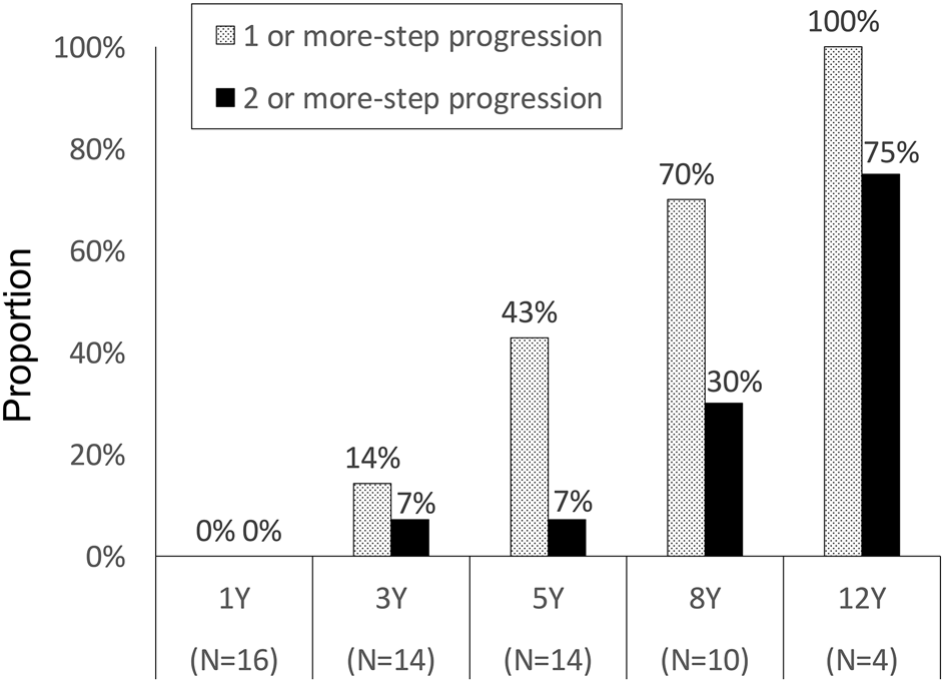
Proportion of patients showing the 1-step or more (dotted square) or 2-step (solid square) progression of the grade compared with the initial assessment at 1, 3, 5, 8, or 12 years.

According to the Simple Classification system, the initial examination revealed 4, 7, 5, or 0 eyes classified into grade 0, 2, 3, or 4, respectively. Likewise, at the last examination, 3, 4, 5, or 4 eyes were classified into the same grades. Figure 1 illustrates a representative case of MacTel type 2 in the current cohort. None of the eyes exhibited exudative MNV (grade 6) during the follow-up period, although nonexudative MNV was observed in 2 eyes of one patient. FTMH was observed in two eyes, with one eye developing FTMH 3 years after the initial visit. Subsequently, pars plana vitrectomy was performed, resulting in the closure of the hole. No patients underwent treatments such as laser therapy or administration of antiangiogenic agents during the follow-up period. The longitudinal changes in the grade on the Simple Classification system are depicted in Fig. 2. At year 1, the grades of each eye were similar to the baseline. However, at year 3 or longer, a number of eyes exhibited progression from grade 0–3 to grade 2–4. The proportion of eyes showing progression of 1 or more steps or 2 or more steps in grade after 1, 3, 5, 8, and 12 years was 0%, 14%, 43%, 70%, and 100%, or 0%, 7%, 7%, 30%, and 75%, respectively (Fig. 3). Notably, no eyes exhibited grade 5 (central pigment) or 6 (exudative MNV) at the initial visit or at any subsequent visits. Furthermore, among the 8 patients, 5 (63%) displayed grade 2 (central EZ break) or more lesions bilaterally, while the remaining 3 (37%) patients exhibited asymmetrical manifestation, with one eye classified as grade 0 and the other eye classified as grade 2 or more.

### Visual outcome

**Figure 4:**
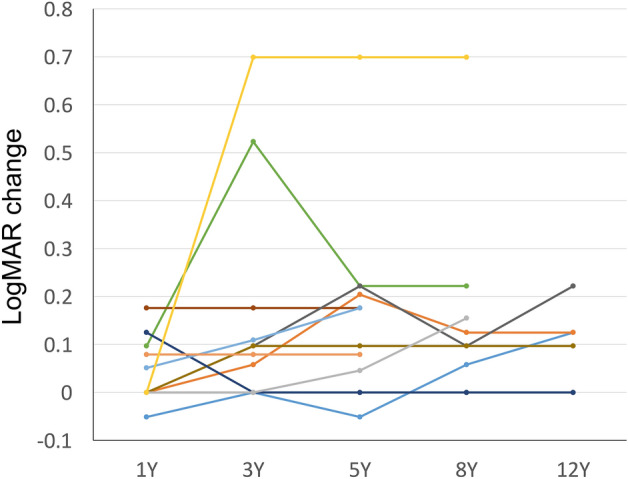
LogMAR change of each eye (n = 16) at the baseline, 1, 3, 5, 8, or 12 years. Of note, the two eyes showing severe visual decline (indicated with yellow and green lines) developed full-thickness macular hole before visual decline.

**Figure 5:**
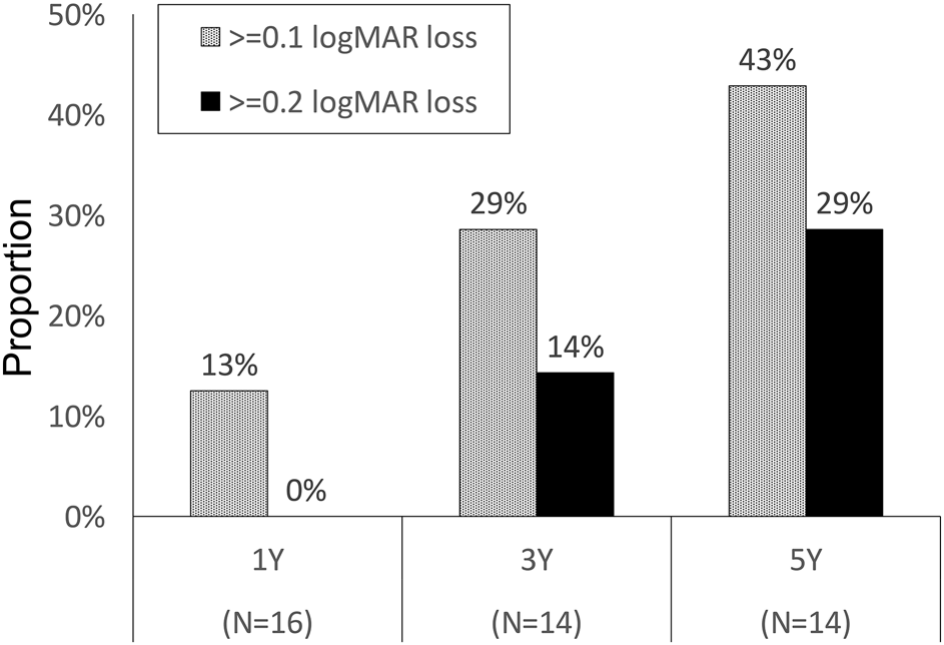
Proportion of the patients showing ≥ 0.1 or ≥ 0.2 logMAR loss after 1, 3, or 5 years.

Regarding the visual outcome, the mean logarithm of minimum angle of resolution (logMAR) was 0.09 ± 0.20 at the initial visit and 0.23 ± 0.32 at the last visit, indicating a significant deterioration (*p* = 0.006, Table 2). Figure 4 illustrates the longitudinal changes in logMAR for each eye. The proportion of eyes showing a visual decline of 0.1 logMAR or more or 0.2 logMAR or more after 1, 3, and 5 years was 13%, 29%, and 43%, or 0%, 14%, and 29%, respectively (Fig. 5). At the last visit, one patient had both eyes with 0.4 logMAR (equivalent of 20/50) or worse, and none had 0.1 logMAR (equivalent of 20/200) or worse in both eyes. Visual prognosis tended to be distinct between eyes with and without FTMH. The eyes with FTMH showed a moderate or severe decrease of 0.3 logMAR or greater (indicated by the yellow and green lines in Fig. 4). In those without FTMH, visual decline was not clearly observed in the short-term but a mild decrease (within 0.3 logMAR) was seen in the long-term follow-up. There was a positive association between grade progression and the proportion of eyes with a visual change of 0.1 logMAR or greater or 0.2 logMAR or greater (*p* = 0.020 or 0.031, respectively).

### Blood test

Blood test results were available for 7 patients (Table 1). Abnormally high serum triglyceride levels or low serum serine levels were observed in 2 or 3 patients, respectively. Three out of the 7 patients showed low serum serine concentration, without any apparent correlation with anatomical or visual outcomes.

## Discussion

In this current study, we investigated the longitudinal anatomical progression and visual outcomes in patients with MacTel type 2 over an average follow-up period of 8 years. Using the Simple Classification system, we observed disease progression in multiple eyes during this time. At the last visit, there was a significant decline in mean visual acuity, and the proportion of eyes experiencing visual loss increased with the length of the follow-up period.

In the current analysis, Simple MacTel Classification system^11^ was used to assess disease progression because it provides information on the rate of progression within 1–5 years for each grade, as well as the association between grade progression and visual loss. Previous research^11^ indicated that 1-step or 2-steps progression over 5 years occurred in approximately 30–40% or 5–25%, respectively, of eyes classified as grade 0, 2, or 3, where the eyes in the current study were classified at the initial examination. It was compatible with the findings of our study, showing 1 or more or 2 or more progression in 5 years was 43% or 7%, respectively. These results suggest that disease progression in this population can be similar according to Simple Classification system. Additionally, our study revealed the progression over 8 or 12 years increased with time, suggesting the disease progression may not slow down even after a decade.

Furthermore, the present study showed that 37% of patients exhibited asymmetrical manifestation. Although MacTel 2 is usually bilateral, some patients showed asymmetricity^14,15^. Clemons et al. reported in the large-scale cohort study that many of the image characteristics and visual acuity had small to moderate agreement between eyes^16^.

As for visual acuity, a large-scale observational study among MacTel Project revealed that, after 5 years, there was a 27% probability of losing 10 or more letters, and a 15% probability of losing 15 or more letters^17^. Conversely, a previous case series involving 16 eyes of 8 Japanese patients^13^ indicated a more favorable visual outcome, with either improvement or maintenance during the follow-up period, although the mean follow-up time was less than 2 years. In our current study, which had a mean follow-up time of 8 years, it was observed that visual acuity significantly declined at the last examination, and 29% of eyes exhibited a decline of 0.2 logMAR or more after 5 years. These findings align with the previous report^17^. The current study also revealed that as the disease grade progressed, a greater proportion of eyes experienced visual loss, consistent with previous findings by Chew et al.^11^. These results suggest a similar outcome to the MacTel Project cohort^16^, which primarily comprised of Caucasian participants, with regards to visual changes over a longitudinal observation for 5 years or more. However, additional research involving a larger population of Asian individuals is necessary to validate this hypothesis. It is worth noting that the visual prognosis appeared to differ between eyes with and without FTMH. Eyes with FTMH exhibited moderate or severe visual decline (0.3 logMAR or greater), consistent with findings from a cross-sectional multicenter study^18^, which reported the possibility of severe vision loss in eyes with FTMH. Conversely, eyes without FTMH did not demonstrate significant visual decline, but did exhibit mild deterioration (within 0.3 logMAR) during the long-term follow-up, consistent with a previous report^10^.

In this study, we observed a decrease in serum serine levels in three patients with no apparent clinical differences, including the rate of progression, between eyes with decreased serine levels and those with normal levels. To the best of our knowledge, there have been no reports investigating the association between serum serine levels and longitudinal clinical course. Previous studies have suggested an association between abnormalities in serine-glycine metabolism and the onset of MacTel type 2, with the underlying abnormal synthesis of deoxysphingolipids being essential^19^. Deoxysphingolipids are known to exhibit neurotoxicity and have been shown to negatively correlate with serine levels in the serum of MacTel type 2 patients^8^. Therefore, monitoring the levels of deoxysphingolipids that may be more directly involved in the pathophysiology of MacTel type 2 is considered helpful.

The study has several limitations. It was a retrospective nonrandomized design, and the numbers of cases were relatively small. There might be some biases with a limited number of subjects in the current analysis and further analysis with a much greater number of patients by, for example, a multicenter study is required to confirm the findings from the current analysis. The follow-up time of each patient was variable, and retinal function was assessed only with visual acuity, not a microperimetric test. Therefore, a more extensive longitudinal study with a larger sample size will be necessary to validate the present findings.

In conclusion, we performed a longitudinal study to observe clinical characteristics of East Asian patients with MacTel type 2 over an average follow-up duration of 8 years. Our findings revealed significant progression in visual acuity and disease grade, which correlated with the length of the follow-up period. Notably, cases that developed FTMH demonstrated a substantial decline in visual acuity, while cases without FTMH experienced a noticeable but mild deterioration. Moreover, our analysis did not show any apparent association between serum serine levels and clinical progression.

## Methods

### Design

This retrospective study was conducted in accordance with the tenets of the Declaration of Helsinki and was approved by the institutional review board (IRB) of the University of Tokyo (No. 2217). The requirement for written informed consent was waived by the IRB. However, participants who did not authorize the use of their medical records for research were excluded from the study.

### Participants

We included patients who initially visited the Specialized Outpatient Clinic for Macular Diseases at the University of Tokyo Hospital between May 2005 and May 2021 and were diagnosed with MacTel type 2. They underwent a standard examination that included measurement of best-corrected visual acuity (BCVA), slit-lamp biomicroscopy, color fundus photography (CFP) using TRC 50DX retinal camera (Topcon, Tokyo, Japan), and spectral domain optical coherence tomography (SD-OCT) using 3D-OCT 1000 (Topcon, Tokyo, Japan) or Spectralis OCT (Heidelberg Engineering, Heidelberg, Germany). Fundus autofluorescence imaging and blue light reflectance were acquired with Heidelberg Retina Angiograph 2 (Heidelberg Engineering, Heidelberg, Germany). BCVA was measured using the Landolt C chart, and values were converted from decimal acuity to logarithm of minimal angle of resolution (logMAR). All patients underwent fluorescein angiography (FA). OCT angiography (RTVue XR Avanti, Optovue Inc., Fremont, CA, USA) was performed to examine the development of macular neovascularization (MNV). Diagnosis of MacTel type 2 was based on characteristic findings on fundoscopy, OCT, and/or FA^2,5^. Multimodal imaging was also used to rule out other diseases, such as diabetic retinopathy, radiation retinopathy, retinal vein occlusion, tamoxifen retinopathy, or macular dystrophy, in order to exclude them from the analysis. The presence of diabetes, hypertension, or dyslipidemia was determined based on the patients' medication. Serum concentrations of triglyceride, total cholesterol, LDL cholesterol, serine, glycine, alanine, and beta-alanine were reviewed from the medical chart.

### Evaluation of anatomical findings

To evaluate the anatomical findings obtained through multimodal imaging, we utilized the Simple MacTel Classification system developed by Chew EY and coworkers in 2023 as part of the MacTel Project^11^. In the system, three anatomical findings were featured as key for classification: OCT hyper-reflectivity, pigment, and EZ break. The severity of the grade is determined based on the decline in BCVA. The system consists of six grades; grade 0 (no lesions), grade 1 (noncentral EZ break), grade 2 (central EZ break), grade 3 (noncentral pigment), grade 4 (OCT hyper-reflectivity), grade 5 (central pigment), and grade 6 (neovascularization with exudative changes). Annual relative risk of progression over 5 years for visual acuity and for progression along the scale were also reported^11^. Additionally, we assessed the development of Full-thickness macular hole (FTMH) and MNV using SD-OCT and OCTA.

### Statistical analysis

We analyzed various demographic factors including age, sex, systemic comorbidities, and laboratory data. The logMAR and the grades on the Simple Classification System were longitudinally investigated. The logMAR changes between the initial and final examinations were analyzed using a mixed model. We also analyzed the association between grade progression and the proportion of eyes with visual changes of 0.1 logMAR or greater, or 0.2 logMAR or greater, from the initial to the last visit using Cochran-Armitage trend analysis. JMP Pro version 16 (SAS Institute Inc.) was used for the statistical analysis. Statistical significance was set at *p* < 0.05.

### Supplementary Information

Supplementary Figure S1.

## Data Availability

The datasets generated during and/or analysed during the current study are available from the corresponding author on reasonable request.
