# Supplementary material for: Longitudinal anatomical and visual outcome of macular telangiectasia type 2 in Asian patients
Source: Sci Rep. 2023 Nov 2;13:18954. doi: 10.1038/s41598-023-46394-4 (PMC10622519; doi:10.1038/s41598-023-46394-4)

Longitudinal anatomical and visual outcome of macular telangiectasia type 2 in Asian patients

Kiyoto Totsuka[1], Shuichiro Aoki[1], Takahiro Arai[1], Kodai Kitamoto[1], Keiko Azuma[1], Ryosuke Fujino[1], Tatsuya Inoue[1,2], Ryo Obata[1*]

1 Department of Ophthalmology, the University of Tokyo Graduate School of Medicine, 7-3-1 Hongo, Bunkyo-ku, Tokyo 113-8655, Japan

2 Department of Ophthalmology and Micro-Technology, Yokohama City University, 4-57 Urafune, Minami-ku, Yokohama, Kanagawa, 232-0024, Japan.

Supplementary Figure S1. Fundus photograph and OCT B-scan with infrared fundus image with Spectralis OCT of both eyes in cases 1 to 8.

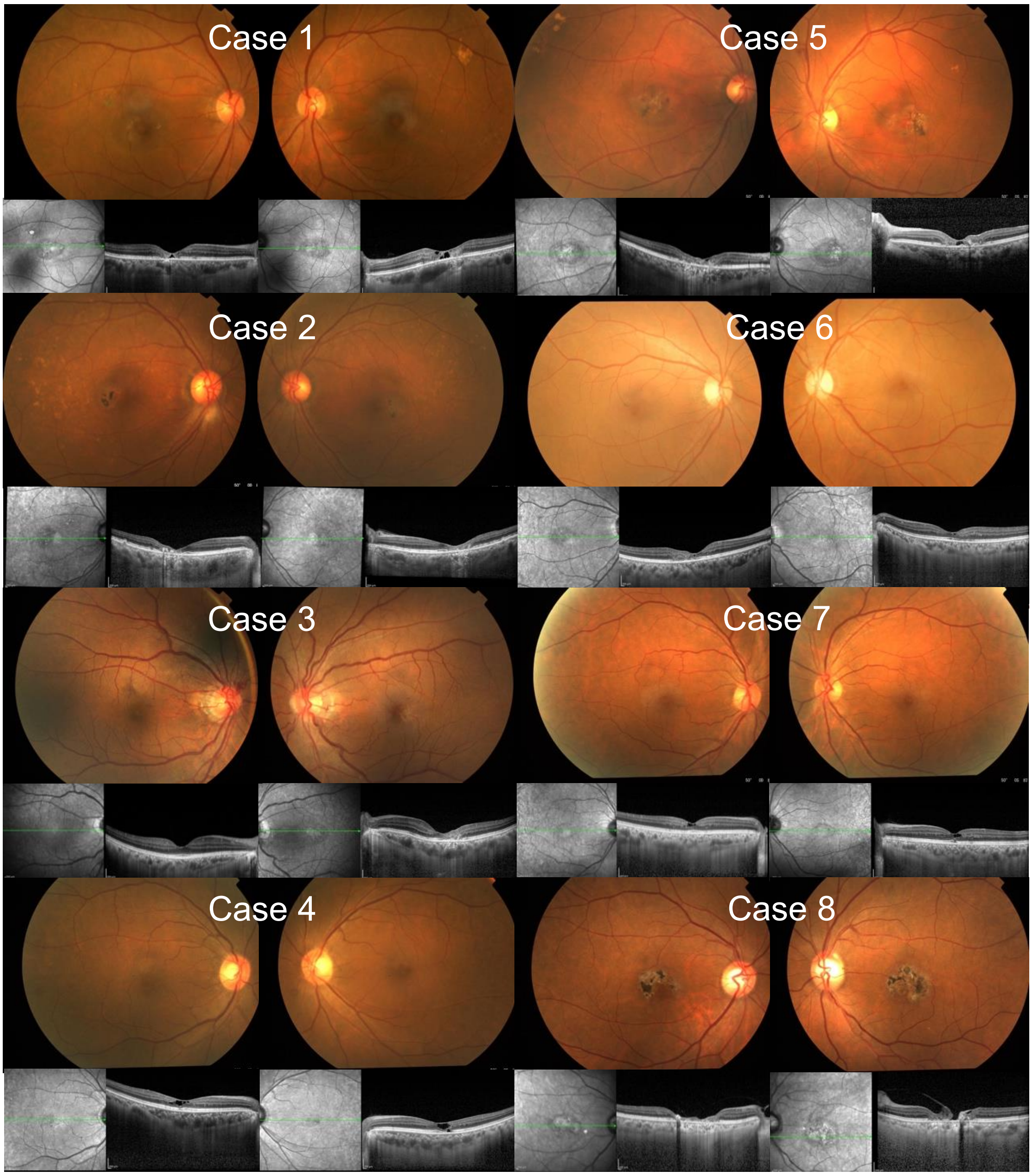

Supplement: Supplementary file 1 — Supplementary Figure S1. [file 41598_2023_46394_MOESM1_ESM.pdf]
